# Broadly Reactive Influenza Antibodies Are Not Limited by Germinal Center Competition with High-Affinity Antibodies

**DOI:** 10.1128/mBio.01859-20

**Published:** 2020-11-03

**Authors:** Rachael Keating, Jenny L. Johnson, David C. Brice, Jocelyn G. Labombarde, Alexander L. Dent, Maureen A. McGargill

**Affiliations:** a Department of Immunology, St. Jude Children’s Research Hospital, Memphis, Tennessee, USA; b Department of Microbiology and Immunology, Indiana University School of Medicine, Indianapolis, Indiana, USA; University of Colorado School of Medicine

**Keywords:** antibody repertoire, humoral immunity, influenza, influenza vaccines

## Abstract

Enhancing the generation of broadly reactive antibodies against influenza A virus (IAV) is a pertinent goal toward developing a universal IAV vaccine. While antibodies that bind conserved IAV epitopes have been identified in humans, antibodies specific for the variable epitopes are much more prevalent than antibodies recognizing conserved epitopes. It is important to define the factors that limit the generation of broadly reactive IAV antibodies in order to develop an effective universal IAV vaccine. The predominant theory is that competition within germinal centers favors the synthesis of high-affinity antibodies specific for the variable region of the virus, and limits antibodies specific for conserved IAV epitopes. Here, we show that reducing germinal center formation and removing competition with high-affinity antibodies was not sufficient to increase broadly reactive IAV antibodies or enhance protection against distinct IAV subtypes. These data disprove the prevailing hypothesis that broadly reactive IAV antibodies are rare due to competition within germinal centers, and reveal the critical need to further investigate factors that limit broadly reactive IAV antibodies. Additionally, our data show that IAV-specific IgM antibodies persist in mice in the absence of germinal centers, highlighting the protective capacity of germinal center-independent IgM antibodies, which are not typically considered when testing correlates of protection, and offer an alternate target for delivering a universal IAV vaccine.

## Abstract (precis)

It is estimated that 250,000 to 650,000 individuals worldwide die each year from seasonal influenza A virus (IAV) infections. Current vaccines provide little protection against newly emerging strains. Thus, considerable effort is focused on enhancing the generation of broadly reactive IAV antibodies in order to develop a universal IAV vaccine. However, broadly reactive IAV antibodies are rare and the factors that limit their generation are not completely understood. Our data disprove the prevailing hypothesis that broadly reactive IAV antibodies are uncommon due to competition in the germinal centers with antibodies specific for the variable, hemagglutinin (HA) head. Understanding the factors that constrain development of antibodies specific for conserved regions of IAV is imperative for developing an effective universal IAV vaccine, which could potentially circumvent a catastrophic pandemic. These findings are significant as they highlight the importance of investigating other mechanisms that contribute to the paucity of broadly reactive IAV antibodies.

## INTRODUCTION

Seasonal influenza A virus (IAV) vaccines do not effectively protect against novel IAV strains that emerge each year. Consequently, there is considerable interest in developing a universal IAV vaccine that induces immunity to epitopes conserved across different IAV subtypes, thereby providing long-lasting, heterosubtypic protection against multiple influenza strains. The antibody response to IAV is dominated by antibodies specific for the globular head domain of one of the surface glycoproteins, hemagglutinin (HA) (1). Antibodies targeting the HA head neutralize IAV by preventing the virus from binding host epithelial cells. To escape immune detection, the IAV mutates key residues of the HA head region, which frequently gives rise to novel strains. Thus, the most effective antibodies generated by current vaccines are specific for the most variable region of the virus, and therefore only provide strain-specific protection. In addition to the variable HA head, broadly reactive IAV antibodies specific for conserved epitopes, including those in the membrane proximal stalk region of HA, have been identified in humans (2–5). Broadly reactive IAV antibodies have the capacity to protect against infection with multiple IAV subtypes and, therefore, form the basis of a universal vaccine. However, these “broad spectrum” B cell clones are extremely rare and consequently do not contribute significantly to the antibody response following vaccination (1, 6–9). Indeed, it is estimated that antibodies specific for the HA head are 1,000-fold more prevalent than antibodies targeting conserved epitopes (10). A better understanding of factors limiting antibodies specific for conserved regions of the IAV is critical for developing a universal vaccine, which could potentially circumvent a pandemic.

Following IAV infection or vaccination, naive B cells encounter antigen in the draining lymph nodes or the spleen. IAV-specific B cells are activated and can differentiate into short-lived plasma cells, memory B cells, or seed germinal centers (11). B cells with the highest avidity for antigen are selected to develop in germinal centers through a series of interactions with T follicular helper cells (T_FH_), which provide essential factors that promote proliferation and further hypermutation of B cells. As T_FH_ are limiting, B cell receptors (BCRs) with the highest affinity have a competitive advantage over lower-affinity BCRs (11). Following affinity maturation in the germinal center, B cells can differentiate into antibody-secreting plasma cells or memory B cells.

Although it is well established that the B cell response following IAV infection or vaccination is heavily biased toward epitopes in the variable head region of HA relative to conserved HA stalk epitopes, the mechanisms that mediate this immunodominance are not completely understood (12). Several factors contribute to the scarcity of broadly reactive antibodies, including steric hindrance of conserved epitopes, antigen quantity, naive B cell precursor frequency, B cell receptor avidity, and immunization route (11–14). Although high-affinity antibodies can develop outside germinal centers, the germinal center reaction significantly shapes the antibody repertoire by promoting the development of high-affinity antibodies over antibodies with a lower affinity. Thus, the prevailing hypothesis for the paucity of broadly reactive IAV antibodies is that antibodies specific for the conserved epitopes are out-competed in germinal centers by high-affinity antibodies specific for the HA head region (11, 12). This is supported by the finding that immunization with the conserved HA stalk region, in the absence of the variable HA head, can elicit a robust, high-affinity antibody response, suggesting that eliminating competition by head-specific antibodies allows development of broadly reactive IAV antibodies (14, 15). Additionally, mice treated with a low dose of rapamycin during IAV infection had impaired germinal center formation, reduced IAV-specific IgG, and an unexpected increase in broadly reactive antibodies that protected mice against subsequent heterosubtypic infections (16). These data support the predominant theory that competition in germinal centers is a major factor limiting development of antibodies specific for conserved IAV epitopes. Moreover, these findings imply that reducing high-avidity antibodies specific for variable HA epitopes could permit the expansion of broadly reactive IAV antibodies, and consequently increase heterosubtypic immunity. Therefore, we tested whether inhibiting germinal center formation was sufficient to limit high-avidity IAV-specific antibodies and increase production of broadly reactive IAV antibodies. We found that blocking germinal center formation impaired the synthesis of high-avidity IgG antibodies. However, removing germinal center competition was not sufficient to increase the prevalence of antibodies specific for the conserved IAV epitopes, or increase heterosubtypic protection following secondary infection. Importantly, we also discovered that heterosubtypic protection correlated with an increase in IAV-specific IgM antibodies. These data demonstrate that B cell competition in germinal centers is not the main determinant responsible for immunodominance or for the paucity of broadly reactive IAV antibodies elicited against conserved epitopes. Further, these findings demonstrate the importance of investigating other factors that may be limiting the development of broadly reactive IAV antibodies.

## RESULTS AND DISCUSSION

### Inhibition of germinal centers reduces IAV-specific IgG but not IgM antibodies.

To test the hypothesis that the antibody response to conserved regions of IAV is limited by competition within germinal centers, we utilized two different methods to reduce germinal center formation and thwart synthesis of high-avidity IgG antibodies. In the first model, germinal centers were blocked by administering anti-CD40L blocking antibody on days 6 and 8 following infection (17, 18). For the second model, germinal centers were reduced by conditional deletion of *Bcl-6* mediated by CD4-Cre (*Bcl6^fl/fl^*·*CD4-Cre; Bcl6^CD4^*). It was previously demonstrated that *Bcl6^CD4^* mice have normal B cell and T cell development, but lack T_FH_ and germinal centers following immunization (19). Importantly, the effector T cell response to immunization is largely intact in *Bcl6^CD4^* mice. In both models, mice were injected intraperitoneally (i.p.) with an H3N2 strain of influenza (A/HK/x31 [X-31]). When given i.p., IAV undergoes limited replication, yet produces the full spectrum of proteins, which is a well-established model of vaccination (20, 21). We previously showed that mice treated with a low dose of rapamycin, during IAV infection, had reduced numbers of germinal centers and an increase in broadly reactive IAV antibodies, which protected mice from subsequent heterosubtypic infections (16). Therefore, we also treated mice daily with rapamycin (or PBS as a control) to compare whether germinal center formation was reduced to a similar extent by anti-CD40L or elimination of T_FH_ cells.

**FIG 1:**
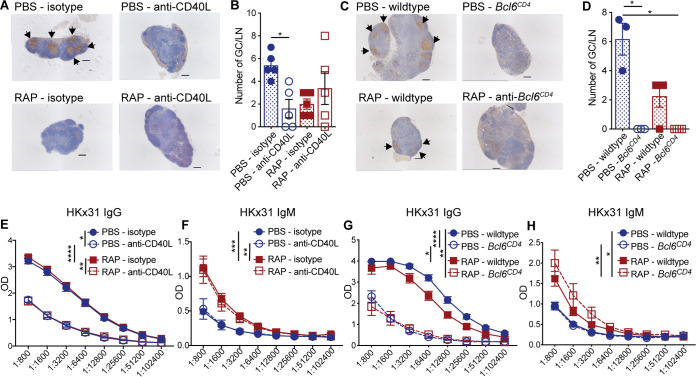
Inhibition of germinal centers reduces influenza-specific IgG, but not IgM, antibodies. (A) C57BL/6 mice were infected i.p. with X-31 and treated daily with rapamycin or PBS as a control. Mice were also given anti-CD40L or isotype control at 6 and 8 days following immunization. Mediastinal lymph nodes were removed 15 days after immunization and sections were stained with anti-Bcl6 to identify germinal centers (indicated by arrowheads). (B) The average number of germinal centers per lymph node is depicted. Data are representative of two independent experiments. Kruskal Wallis test; *, *P* < 0.05; mean ± standard error of the mean (SEM) of *n* = 5. (C) Wild-type or *Bcl6^CD4^* mice were infected i.p. with X-31 and treated daily with rapamycin or PBS. Mediastinal lymph nodes were removed 15 days after immunization and analyzed for germinal centers. (D) The average number of germinal centers per lymph node is depicted. Data are representative of one experiment. Kruskal Wallis test; *, *P* < 0.05; mean ± SEM of *n* = 3 or 4. (E and F) Serum from anti-CD40L or isotype-treated mice were taken 28 days after immunization and analyzed by ELISA for IgG (E) or IgM (F) antibodies specific for X-31. Freidman test with Dunn’s multiple-comparison test; *, *P* < 0.05; **, *P* < 0.01; ***, *P* < 0.001; ****, *P* < 0.0001; mean ± SEM of *n* = 7 to 10. Data are representative of two independent experiments. (G and H) Serum from wild-type or *Bcl6^CD4^* mice was taken 28 days after immunization and analyzed by ELISA for IgG (G) or IgM (H) antibodies specific for X-31. Data are representative of two experiments. Freidman test with Dunn’s multiple-comparison test; *, *P* < 0.05; **, *P* < 0.01; ****, *P* ≤ 0.0001; mean ± SEM of *n* = 11 to 15.

To assess the extent of germinal center formation, the mediastinal lymph nodes (MLN) that drain the peritoneal cavity were removed 15 days after exposure to X-31 and analyzed for germinal centers by immunohistochemistry staining for Bcl-6. Anti-CD40L blockade reduced germinal center formation to a level comparable to daily rapamycin treatment (Fig. 1A and B). Similarly, *Bcl6^CD4^* mice had a profound defect in germinal center formation compared to wild-type controls (Fig. 1C and D). Although germinal center numbers were lower in *Bcl6^CD4^* mice compared to mice treated with rapamycin or anti-CD40L, B cells expressing Bcl-6 were still identifiable, but not organized into germinal centers (Fig. 1C). Together, these data show reduced germinal center formation following exposure to IAV via three distinct mechanisms: anti-CD40L treatment, T_FH_ elimination, and rapamycin treatment.

To determine the impact of reducing germinal center formation on the responding antibody response, we analyzed sera for X-31-specific IgG and IgM at 28 days following administration of X-31. Mice given anti-CD40L antibody had reduced X-31-specific IgG levels, but X-31-specific IgM levels were maintained compared to isotype controls (Fig. 1E and F). Similarly, in *Bcl6^CD4^* mice, X-31-specific IgG, but not IgM, was reduced compared to wild-type controls (Fig. 1G and H). These data are consistent with previous findings that long-lived IgM, but not IgG, plasma cells develop in the absence of germinal center formation (22). Interestingly, although germinal centers were reduced in rapamycin-treated mice compared to controls, the amount of X-31-specific IgG was similar between rapamycin and PBS-treated mice (Fig. 1E and G). Moreover, as we previously reported, rapamycin-treated mice had more X-31-specific IgM compared to control mice. However, the increase in X-31-specific IgM was not observed in *Bcl6^CD4^* mice or mice treated with anti-CD40L compared to controls (Fig. 1F and H). It is intriguing that germinal centers were reduced comparably in rapamycin-treated, anti-CD40L-treated, and *Bcl6^CD4^* mice, yet only the rapamycin-treated mice displayed increased levels of X-31-specific IgM, without a reduction in IgG. These data suggest that the increased X-31-specific IgM in rapamycin-treated mice is not simply due to a reduction in germinal centers and reduced class switching to IgG, implying that rapamycin impacts the generation of IAV-specific IgM by a different mechanism. Although germinal centers have been typically regarded as the major site of antibody isotype switching, several groups have demonstrated extrafollicular isotype switching (23–26). In fact, Roco et al. recently reported that the majority of isotype switching occurs prior to germinal center formation (27). The fact that IgG is reduced in anti-CD40L-treated and *Bcl6^CD4^* mice, but not in rapamycin-treated mice, suggests that anti-CD40L signals by T_FH_ are required for extrafollicular isotype switching and that rapamycin is modulating a distinct signaling pathway. Together, these results indicate effective methods to limit germinal center formation following IAV exposure and, interestingly, these methods have distinct impacts on the antibody response. As germinal center-independent IgM memory cells can be long-lived, these cells may be good candidates to target for vaccine development (22, 28–31).

### Germinal center inhibition decreases high-avidity H3-specific IgG antibodies.

**FIG 2:**
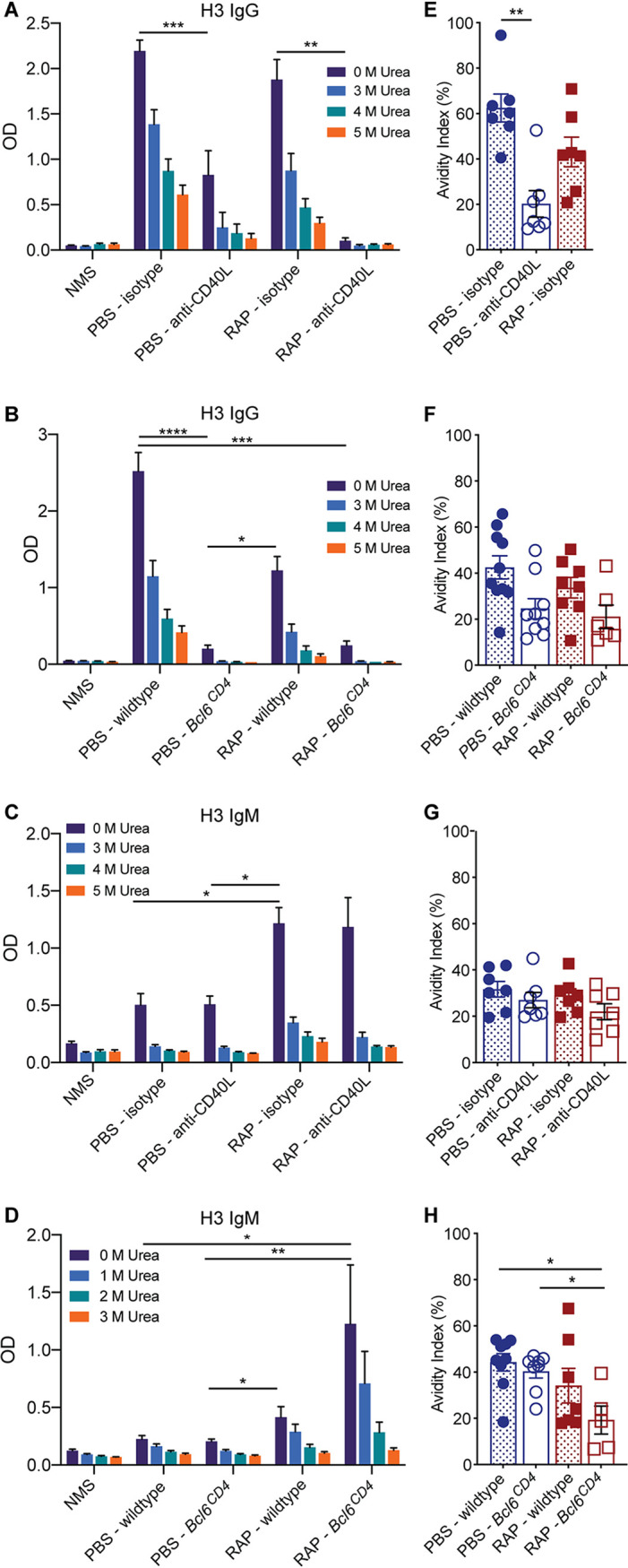
Germinal center inhibition decreases high-avidity H3-specific IgG antibodies. Serum from anti-CD40L or isotype-treated mice was taken 28 days after immunization with X-31 and analyzed by ELISA for H3-specific IgG (A) or H3-specific IgM (C). Serum from wild-type or *Bcl6^CD4^* mice was taken 28 days after immunization and analyzed by ELISA for H3-specific IgG (B) or H3-specific IgM (D). To determine the avidity of the antibodies, urea was added at decreasing concentrations and the avidity indexes were calculated as the ratio of OD at 3 M urea/OD with no urea (Abs 3 M urea/AbsDiluent). (E to H) Avidity indexes for H3 IgG (E and F) and H3 IgM (G and H) are depicted. Data are representative of two independent experiments. Kruskal Wallis test; *, *P* < 0.05; **, *P* < 0.01; ***, *P* < 0.001; ****, *P* ≤ 0.0001; mean ± SEM of *n* = 5 to 10; NMS and Vn1203 positive control, *n* = 4 to 5.

To determine whether the synthesis of high-avidity antibodies specific for HA was impaired in mice with fewer germinal centers, we analyzed sera from mice harvested 28 days after X-31 exposure by an H3 HA enzyme-linked immunosorbent assay (ELISA) with increasing concentrations of urea. Similar to our observations with whole virus, H3-specific IgG levels were reduced in anti-CD40L-treated and *Bcl6^CD4^* mice compared to controls (Fig. 2A and B), while H3-specific IgM levels were maintained (Fig. 2C and D). This suggests that the immunodominance of the HA epitope in shaping the IgM repertoire can be established independent of germinal center competition. Furthermore, in both models, rapamycin-treated mice had more H3-specific IgM relative to PBS-treated control mice (Fig. 2C and D). As expected, the avidity of H3-specific IgG antibodies produced in the anti-CD40L-treated mice was significantly reduced compared to isotype control-treated mice, with a similar trend observed in *Bcl6^CD4^* mice relative to wild-type controls (Fig. 2E and F). These findings are consistent with the established theory that germinal centers are the main site of antigen-driven affinity maturation (11). Interestingly, despite a reduction in germinal centers in mice treated with rapamycin, the level of IgG specific for whole virus was not reduced (Fig. 1E and G); however, there was a trend toward smaller amounts of high-avidity H3-specific IgG antibodies relative to PBS-treated controls (Fig. 2E and F). These data demonstrate that class switching to IgG occurs outside germinal centers, and further highlight the role of germinal centers in fine-tuning the avidity of IgG to specific epitopes. In contrast to the IgG antibodies, the absence of germinal centers did not reduce the avidity of H3-specific IgM compared to controls, except for rapamycin-treated *Bcl6^CD4^* mice (Fig. 2G and H). In general, the avidity of the H3-specific IgM antibodies was reduced in comparison to H3-specific IgG, as indicated by the greater impact of urea on IgM binding compared to IgG antibodies (Fig. 2E and H). Together, these data indicate that inhibiting germinal center formation, via anti-CD40L or in *Bcl6^CD4^* mice, effectively reduced the prevalence of high-avidity IAV-specific IgG antibodies.

### Eliminating germinal center competition does not promote broadly reactive IAV antibodies.

**FIG 3:**
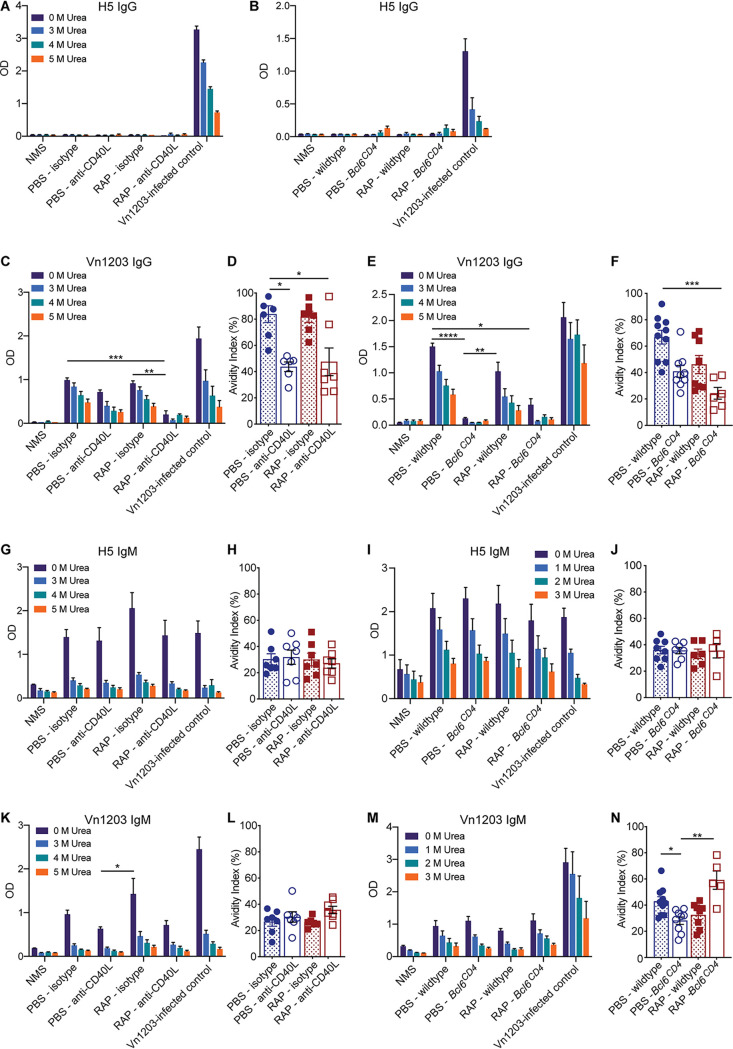
Reducing high-avidity H3-specific IgG antibodies does not promote influenza cross-protective antibodies. Sera from anti-CD40L and isotype-treated mice (A) or wild-type and *Bcl6^CD4^* mice (B) were taken 28 days after immunization with X-31 and analyzed by ELISA for H5-specific IgG (A and B), ΔVn1203-specific IgG (C to F), H5-specific IgM (G to J), or ΔVn1203-specific IgM (K to N). Avidity indexes were determined with 3 M urea (D, F, H, J, and L) or 2 M urea (N). Data are representative of at least two independent experiments. Kruskal Wallis test; *, *P* < 0.05; **, *P* < 0.01; ***, *P* < 0.001; mean ± SEM of *n* = 5 to 10 for the samples; NMS and Vn1203 positive control, *n* = 3 to 5.

To determine whether reducing germinal center formation and the frequency of high-avidity H3-specific IgG antibodies gives rise to an increase in broadly reactive IAV antibodies, we tested sera from mice given X-31 (H3N2) for antibodies specific for an H5 HA protein from strain A/Vietnam/1203/04 (Vn1203) or a recombinant H5N1 virus that contains H5 and N1 from Vn1203 in combination with the same internal genes as X-31 (ΔVn1203). The frequency of broadly reactive, H5-specific IgG antibodies was negligible for all groups of mice (Fig. 3A and B). IgG antibodies specific for the whole ΔVn1203 virus were detectable in mice given X-31, which may include antibodies to conserved epitopes on the HA protein as well as antibodies to conserved epitopes on other proteins, including the internal proteins. However, the reduction in high-avidity anti-H3 IgG antibodies in mice treated with anti-CD40L did not lead to an increase in broadly reactive IgG antibodies compared to control mice (Fig. 3C). Likewise, *Bcl6^CD4^* mice did not have an increase in broadly reactive IgG compared to wild-type mice (Fig. 3E). Not surprisingly, given the diminished opportunity for germinal center affinity maturation, the avidity of the cross-reactive, ΔVn1203-specific IgG antibodies was reduced in anti-CD40L-treated and *Bcl6^CD4^* mice compared to control mice (Fig. 3D and F). Although all groups of mice lacked IgG antibodies specific for H5, all mice given X-31 had an increase in H5-specific IgM antibodies compared to naive mice (NMS) (Fig. 3G and I). Likewise, IgM antibodies specific for the whole ΔVn1203 virus increased in all mice exposed to X-31 relative to uninfected mice (Fig. 3K and M). There was a trend toward increased H5 and ΔVn1203-specific IgM in rapamycin-treated mice compared to PBS controls, but not in mice treated with anti-CD40L (Fig. 3G and K). However, reducing high-avidity antibodies in anti-CD40L-treated or *Bcl6^CD4^* mice did not increase the amount of cross-reactive H5- or ΔVn1203-specific IgM relative to control mice with intact germinal centers (Fig. 3G to M). Interestingly, rapamycin-treated *Bcl6^CD4^* mice that had a reduction in high-avidity H3-specific IgM antibodies (Fig. 2G and H) also showed an increase in high-avidity ΔVn1203-specific IgM antibodies (Fig. 3N). Together, these results indicate that limiting the formation of the high-avidity H3-specific IgG antibodies by eliminating germinal center competition is not sufficient to increase the prevalence of broadly reactive IAV antibodies. Furthermore, rapamycin alters the antibody repertoire via a mechanism independent of germinal center reduction, and this impact is enhanced in *Bcl6^CD4^* mice, which lack T_FH_.

### Limiting high-avidity antibodies via *Aicda* deletion is not sufficient to boost broadly reactive IAV antibodies.

**FIG 4:**
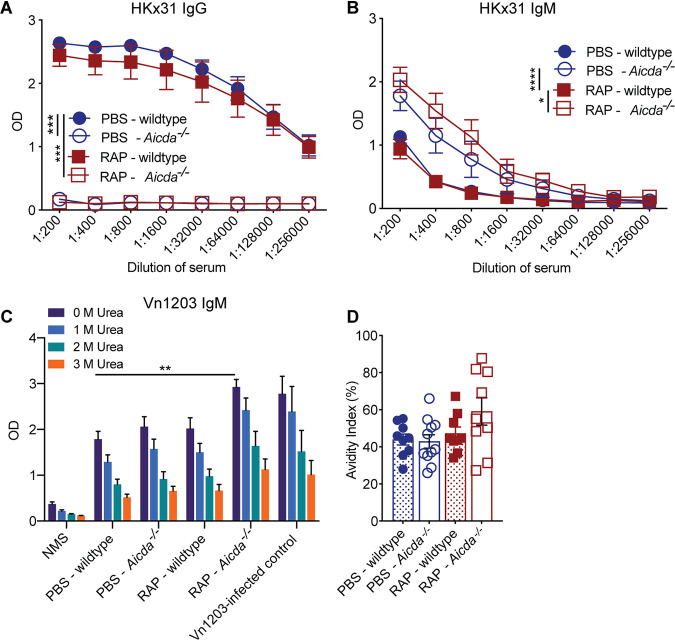
Limiting high-avidity antibodies via *Aicda* deletion is not sufficient to boost influenza cross-protective antibodies. Wild-type or *Aicda^−/−^* mice were infected i.p. with X-31 and treated daily with rapamycin or PBS. Sera were taken 28 days after immunization and analyzed by ELISA for X-31-specific IgG (A) or X-31-specific IgM (B). Freidman test with Dunn’s multiple-comparison test; *, *P* < 0.05; ***, *P* < 0.001; ****, *P*< 0.0001; mean ± SEM of *n* = 6 to 7. Data are representative of one experiment. (C) Serum from mice immunized 28 days prior with X-31 was analyzed for ΔVn1203-specific IgM antibodies with increasing concentrations of urea. Kruskal Wallis test; **, *P* < 0.01. (D) The avidity index was calculated with 3 M urea. Data are representative of one experiment. Mean ± SEM of *n* = 9 to 11; NMS and ΔVn1203-infected control, *n* = 3 to 6.

Our study highlights that IAV-specific IgM and IgG antibodies can be maintained independently of germinal centers. It is possible that germinal center-independent IgM antibodies are limited by IgG antibodies that are generated outside the germinal center. Additionally, within germinal centers, broader IgM repertoires may develop in the absence of high-avidity antibodies. Therefore, we sought to limit high-avidity antibodies while keeping the germinal centers intact. To this end, we administered X-31 to *Aicda*^−/−^ mice, which cannot undergo somatic hypermutation or isotype class switching, and therefore cannot produce IgG or high-avidity antibodies either in germinal centers or outside germinal centers (32). The *Aicda*^−/−^ and wild-type C57BL/6 mice were injected i.p. with X-31, and antibodies in the sera were analyzed 28 days later. As expected, *Aicda*^−/−^ mice did not have any X-31-specific IgG in either rapamycin- or PBS-treated mice (Fig. 4A). In addition, the level of X-31-specific IgM increased in *Aicda*^−/−^ mice treated with rapamycin or PBS compared to wild-type mice, which likely reflects accumulation of IgM due to a block in class switching to IgG (Fig. 4B). Similar to anti-CD40L-treated or *Bcl6^CD4^* mice lacking germinal centers, *Aicda*^−/−^ mice that lack high-avidity IgG did not have an increase in broadly reactive IgM antibodies relative to wild-type mice (Fig. 4C), suggesting that removal of the high-avidity IgG antibodies was not sufficient to increase broadly reactive antibodies. Remarkably, *Aicda*^−/−^ mice treated with rapamycin had the highest levels of ΔVn1203-specific IgM antibodies and the highest-avidity antibodies compared to all other groups of mice (Fig. 4D), which was similar to rapamycin-treated *Bcl6^CD4^* mice. Together, these data confirm that removing high-avidity H3-specific IgG antibodies is not sufficient to promote the generation of broadly reactive IAV antibodies. Additionally, rapamycin enhances the generation of broadly reactive antibodies via a mechanism other than removing competition from high-avidity antibodies.

### Reducing development of strain-specific IAV antibodies is not sufficient to boost immunity to subsequent heterosubtypic IAV infection.

**FIG 5:**
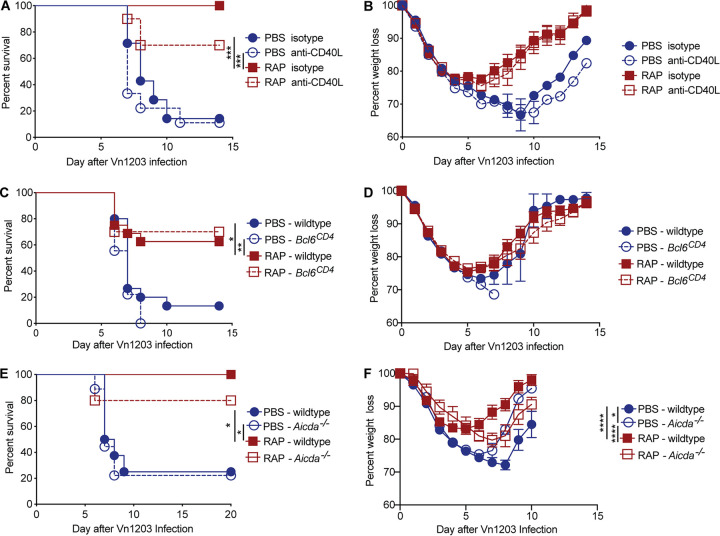
Reducing high-avidity IgG antibodies is not sufficient to boost immunity to subsequent heterosubtypic influenza infections. (A and B) C57BL/6 mice treated with anti-CD40L or isotype control antibody were infected with ΔVn1203 i.n. at 30 days after X-31 immunization and daily injections of PBS or rapamycin. Mice were monitored for survival (A) and weight loss (B). Data are representative of two independent experiments, *n* = 7 to 10. (C and D) Wild-type and *Bcl6^CD4^* mice were infected with ΔVn1203 i.n. at 30 days after X-31 immunization and daily injections of PBS or rapamycin. Mice (*n* = 9 to 17/group) were monitored for survival (C) and weight loss (D). Data are representative of three independent experiments. (E and F) C57BL/6 mice and *Aicda^−/−^* mice were infected with ΔVn1203 i.n. at 30 days after X-31 immunization and daily injections of PBS or rapamycin. Mice were monitored for survival (E) and weight loss (F). Data are representative of two independent experiments (*n* = 5 to 9). Survival curves for PBS (wild type/isotype) versus PBS (knockout/anti-CD40L), PBS (wild type/isotype) versus RAP (wild type/isotype), and PBS (knockout/anti-CD40L) versus RAP (wild type/isotype) were analyzed by Mantel-Cox with Holm-Sidak multiple-comparison test, and weight loss was analyzed by linear mixed-effects model Holm-Sidak multiple-comparison test. *, *P* < 0.05; **, *P* < 0.01; ***, *P* < 0.001; ****, *P* < 0.0001.

Our previous work demonstrated that rapamycin enhanced protection to heterosubtypic IAV infections when given with an H3N2 virus (16). This protection was B cell-dependent and transferred to naive mice via serum, indicating that rapamycin enhanced protection by altering the antibody response. When analyzing the antibody response to the whole virus, there is only a modest increase in the levels of ΔVn1203-specific IgM antibodies in mice treated with rapamycin compared to control mice (Fig. 3). However, we previously demonstrated that rapamycin altered the specificities of both the IgG and IgM antibodies generated after IAV exposure, and there were significant differences in antibodies specific for particular epitopes in the rapamycin- and PBS-treated mice (16). Thus, although mice that lack high-avidity IgG antibodies via germinal center or *Aicda* deletion did not have an increase in the levels of broadly reactive IAV antibodies, altered antibody specificities may enhance heterosubtypic protection. Therefore, we tested whether restricting the production of high-avidity, strain-specific IgG antibodies in mice given X-31 impacted survival following a secondary challenge with an H5N1 virus (ΔVn1203). Mice were injected i.p. with X-31 and treated with either rapamycin or PBS as a control for 28 days. The following day, mice were given an intranasal challenge with ΔVn1203 and monitored for survival and weight loss. As we reported previously, rapamycin treatment significantly increased survival compared to PBS-treated controls (Fig. 5). However, germinal center reduction via anti-CD40L treatment or in *Bcl6^CD4^* mice was not sufficient to enhance protection against a heterosubtypic virus (Fig. 5A to D). Likewise, deletion of high-avidity antibodies in *Aicda*^−/−^ mice did not enhance protection following an H5N1 infection (Fig. 5E and F). These data indicate that simply reducing germinal center formation or high-avidity IgG antibodies is not sufficient to increase broadly reactive IAV immunity. Notably, the mice that received anti-CD40L and *Bcl6^CD4^* mice treated with rapamycin were protected from ΔVn1203 infection at levels similar to rapamycin-treated wild-type mice, even though these mice had minimal X-31-specific or cross-reactive IgG antibodies. This suggests that rapamycin enhances heterosubtypic immunity in a manner independent of germinal center formation and high-avidity IgG antibodies. Furthermore, the fact that wild-type, anti-CD40L-treated, and *Bcl6^CD4^* mice given rapamycin had higher levels of X-31-specific IgM than the PBS-treated cohorts, which was also observed for cross-reactive IgM antibodies, supports the notion that immunity to conserved portions of IAV may be enhanced by increasing broadly reactive IgM antibodies. The protective role of IgM antibodies in rapamycin-treated mice is best evidenced by the survival of *Aicda^−/−^* mice treated with rapamycin, which completely lack IgG antibodies. Further studies to assess the protective capacity of rapamycin through germinal center-independent IgM antibodies may provide valuable insight into enhancing immunity to conserved IAV epitopes. Together, our data show that reducing germinal center formation and limiting the prevalence of high-avidity antibodies is not sufficient to enhance heterosubtypic immunity to IAV.

In contrast to the general dogma that broadly reactive antibodies are rare due to favored selection of high avidity antibodies in germinal centers, our data demonstrate that restricting development of high-avidity IgG antibodies is not sufficient to increase the prevalence of broadly reactive influenza antibodies. The fact that antibodies specific for the conserved regions of IAV did not develop in the absence of high-avidity antibodies implies that other factors limit the development of broadly reactive antibodies. The precursor frequency of broadly reactive B cells was recently shown to be similar to B cells specific for the variable region, indicating that precursor frequency does not limit broadly reactive antibodies (15). Moreover, in the absence of the HA variable region, antibodies specific for the conserved stalk are generated, demonstrating the immunogenic potential of these epitopes (1, 15). During the 2009 H1N1 pandemic, antibodies specific for the HA stalk were much more prevalent in the general population relative to prior years, suggesting that steric hindrance of the conserved epitopes on the intact virus does not completely block the generation of broadly reactive antibodies (33). Recently, it was demonstrated that antibodies specific for the HA stalk region have a higher likelihood to be polyreactive and bind self-antigens compared to antibodies specific for the variable region. (34, 35). Thus, broadly reactive influenza antibodies may be limited by tolerance mechanisms, rather than competition with antibodies specific for the variable region. Our data highlight the importance of further investigating what factors limit the generation of broadly reactive IAV antibodies.

## MATERIALS AND METHODS

### Mice.

Female, 7 to 8-week-old C57BL/6J mice and *CD4-Cre* mice were obtained from the Jackson Laboratory. Mice with loxP-flanked *Bcl6* alleles were previously described (19) and were crossed with *CD4-Cre* mice to generate *Bcl6^fl/fl^*·*CD4-Cre* (*Bcl6^CD4^*) mice that lack *Bcl6* in T cells. As controls, the *Bcl6^CD4^* mice were compared to either *Bcl6^fl/fl^* mice negative for *CD4-Cre*, or *Bcl6^+/+^* mice positive for *CD4-Cre*. *Aicda*^−/−^ mice (32) were generated at St. Jude from frozen sperm obtained from the RIKEN BioResource Center. Female, 7 to 8-week-old *Aicda*^−/−^ mice were used in experiments with age-matched female C57BL/6J mice. All mice were maintained under specific pathogen-free conditions at St. Jude Children’s Research Hospital, and all animal studies were approved by the Institutional Animal Care and Use Committee.

### Virus and infections.

The X-31 and ΔVn1203 viruses were constructed using the eight-plasmid reverse genetics system (36) and contained the six internal genes of the A/Puerto Rico/8/34 (PR8) strain and genes encoding the HA and NA surface proteins from either A/HKx31 (H3N2) or A/Vietnam/1203/04 (H5N1) strains, respectively. Mice were given 1 × 10^8^ of the 50% egg infective dose (EID_50_) of X-31 intraperitoneally (i.p.) diluted in phosphate-buffered saline (PBS). Beginning1 day prior to X-31 administration, mice received daily i.p. injections of 1.5 μg rapamycin (Rapamune; Wyeth) diluted in PBS, or PBS alone as a control. Mice treated with anti-CD40L antibody were given i.p. injections of 200 μg of anti-CD40L, or an isotype control, on days 6 and 8 relative to X-31 injection. For secondary challenge, after 4 weeks of rapamycin or PBS injections, mice were anesthetized with Avertin (2,2,2-tribromoethanol) and challenged intranasally with 4.5 × 10^5^ EID_50_ of ΔVn1203. For mice given X-31 IP, the 50% lethal dose (LD_50_) of this ΔVn1203 strain is ∼4.0 × 10^5^ EID_50._ Mice were monitored daily for weight loss and clinical signs of disease. The humane endpoint used in all experiments was if the mouse became moribund and was defined by any of the following criteria: (i) inability to right itself when placed on its side; (ii) lack of movement when given a gentle nudge; or (iii) difficulty obtaining food or water. Mice determined to be moribund were euthanized via CO_2_ asphyxiation.

### Germinal center analysis.

Mice were euthanized on day 15 following X-31 infection. Mediastinal lymph nodes were removed and fixed in 4% formaldehyde, embedded in paraffin, sectioned, and stained with anti-Bcl6 (sc-858; Santa Cruz) antibodies. Images were acquired with a Nikon TiE microscope equipped with a 10×, 0.3 NA objective, motorized stage, and DS-Ri2 CMOS camera. Image capture, processing, and analysis were performed using NIS Elements software (Nikon Instruments).

### X-31-specific ELISA.

Microtiter plates (Nunc) were coated with lysed whole X-31 diluted in PBS at 100 ng/well overnight at 4°C. Plates were washed and incubated with serum samples for 2 h, then washed and incubated with goat anti-mouse IgG (1030-04; Southern Biotechnology Associates) or goat anti-mouse IgM (1020-04; Southern Biotechnology Associates) for 1 h. The IgG and IgM antibodies were detected using P-nitrophenyl phosphate (Sigma-Aldrich) added for 30 min to 4 h at 25°C and the optical density (OD) was measured at 405 nm in a microplate reader (Molecular Devices).

### H3-, H5-, and Vn1203-specific ELISA with urea.

Microtiter plates (Nunc) were coated with H3 or H5 protein at 35 ng/well for IgG assays or 70 ng/well for IgM assays or lysed whole Vn1203 diluted in PBS at 10 ng/well for IgG assays, or 20 ng/well for IgM assays, overnight at 4°C. Plates were washed three times with 0.5% Tween in PBS and blocked with 2.5% fetal bovine serum (FBS) in PBS for 1 h at 25°C on a shaker. Plates were incubated with serum samples for 1 h on a shaker and washed three times. Dilutions of urea in PBS (1 M to 5 M) were made and added at 25°C, for 15 min, to destabilize antigen-antibody interactions and make a relative comparison of antibody binding avidity. The plates were then washed three times and incubated with either goat anti-mouse IgG Fc-horseradish peroxidase (HRP) (1033-05; Southern Biotechnology Associates) or goat anti-mouse IgM HRP (1140-05; Southern Biotechnology Associates) for 30 min at 25°C on a shaker. Plates were washed three times and TMB (3,3′,5,5′-tetramethylbenzidine) substrate was added in the dark at 25°C for 15 min. The reaction was stopped via addition of 1 N sulfuric acid. Absorbances were measured at 450 and 550 nm in a microplate reader (Molecular Devices). Background signal (550 nm) was subtracted from absorbance (OD_450_) values to give the relative OD. An avidity index (AI) value was calculated for sera using either 2 M or 3 M urea, in accordance with the rate of antigen-antibody destabilization, and compared to no urea or to diluent alone (AbsUREA/AbsDiluent) (37).

### Statistical analysis.

All data were graphed and analyzed using Prism 7.04 software (GraphPad Software), except for analysis of weight loss as described below. Quantitative differences between more than two groups were compared using the Kruskal-Wallis test followed by a Dunn’s multiple-comparison test. ELISA curves with serial dilutions of serum were compared using the Freidman test for repeated measures, followed by a Dunn’s multiple-comparison test. Survival experiments were analyzed by the Kaplan-Meier survival probability estimates. Weight-loss comparisons were made using a generalized linear mixed model analysis. Models were fit with the lme4 R package, with individual mice included as a random effect to correct for the nonindependence of the data. Residual plots were used to ensure homoscedasticity of the residuals. *P* values of less than 0.05 were considered significant.

## References

[1] Krammer F. 2019. The human antibody response to influenza A virus infection and vaccination. Nat Rev Immunol 19:383–397. doi:10.1038/s41577-019-0143-6. 30837674

[2] Corti D, Suguitan AL, Pinna D, Silacci C, Fernandez-Rodriguez BM, Vanzetta F, Santos C, Luke CJ, Torres-Velez FJ, Temperton NJ, Weiss RA, Sallusto F, Subbarao K, Lanzavecchia A. 2010. Heterosubtypic neutralizing antibodies are produced by individuals immunized with a seasonal influenza vaccine. J Clin Invest 120:1663–1673. doi:10.1172/JCI41902. 20389023 PMC2860935

[3] Ekiert DC, Bhabha G, Elsliger M-A, Friesen RHE, Jongeneelen M, Throsby M, Goudsmit J, Wilson IA. 2009. Antibody recognition of a highly conserved influenza virus epitope. Science 324:246–251. doi:10.1126/science.1171491. 19251591 PMC2758658

[4] Dreyfus C, Laursen NS, Kwaks T, Zuijdgeest D, Khayat R, Ekiert DC, Lee JH, Metlagel Z, Bujny MV, Jongeneelen M, van der Vlugt R, Lamrani M, Korse HJWM, Geelen E, Sahin Ö, Sieuwerts M, Brakenhoff JPJ, Vogels R, Li OTW, Poon LLM, Peiris M, Koudstaal W, Ward AB, Wilson IA, Goudsmit J, Friesen RHE. 2012. Highly conserved protective epitopes on influenza B viruses. Science 337:1343–1348. doi:10.1126/science.1222908. 22878502 PMC3538841

[5] Ekiert DC, Wilson IA. 2012. Broadly neutralizing antibodies against influenza virus and prospects for universal therapies. Curr Opin Virol 2:134–141. doi:10.1016/j.coviro.2012.02.005. 22482710 PMC3368890

[6] Ellebedy AH, Krammer F, Li G-M, Miller MS, Chiu C, Wrammert J, Chang CY, Davis CW, McCausland M, Elbein R, Edupuganti S, Spearman P, Andrews SF, Wilson PC, García-Sastre A, Mulligan MJ, Mehta AK, Palese P, Ahmed R. 2014. Induction of broadly cross-reactive antibody responses to the influenza HA stem region following H5N1 vaccination in humans. Proc Natl Acad Sci U S A 111:13133–13138. doi:10.1073/pnas.1414070111. 25157133 PMC4246941

[7] Sui J, Sheehan J, Hwang WC, Bankston LA, Burchett SK, Huang C-Y, Liddington RC, Beigel JH, Marasco WA. 2011. Wide prevalence of heterosubtypic broadly neutralizing human anti-influenza A antibodies. Clin Infect Dis 52:1003–1009. doi:10.1093/cid/cir121. 21460314 PMC3070035

[8] Corti D, Voss J, Gamblin SJ, Codoni G, Macagno A, Jarrossay D, Vachieri SG, Pinna D, Minola A, Vanzetta F, Silacci C, Fernandez-Rodriguez BM, Agatic G, Bianchi S, Giacchetto-Sasselli I, Calder L, Sallusto F, Collins P, Haire LF, Temperton N, Langedijk JPM, Skehel JJ, Lanzavecchia A. 2011. A neutralizing antibody selected from plasma cells that binds to group 1 and group 2 influenza A hemagglutinins. Science 333:850–856. doi:10.1126/science.1205669. 21798894

[9] Wrammert J, Smith K, Miller J, Langley WA, Kokko K, Larsen C, Zheng N-Y, Mays I, Garman L, Helms C, James J, Air GM, Capra JD, Ahmed R, Wilson PC. 2008. Rapid cloning of high-affinity human monoclonal antibodies against influenza virus. Nature 453:667–671. doi:10.1038/nature06890. 18449194 PMC2515609

[10] Angeletti D, Yewdell JW. 2018. Is it possible to develop a “universal” influenza virus vaccine? Outflanking antibody immunodominance on the road to universal influenza vaccination. Cold Spring Harb Perspect Biol 10:a028852. doi:10.1101/cshperspect.a028852. 28663210 PMC6028072

[11] Victora GD, Wilson PC. 2015. Germinal center selection and the antibody response to influenza. Cell 163:545–548. doi:10.1016/j.cell.2015.10.004. 26496601 PMC4623701

[12] Angeletti D, Yewdell JW. 2018. Understanding and manipulating viral immunity: antibody immunodominance enters center stage. Trends Immunol 39:549–561. doi:10.1016/j.it.2018.04.008. 29789196

[13] Lee PS, Wilson IA. 2015. Structural characterization of viral epitopes recognized by broadly cross-reactive antibodies. Curr Top Microbiol Immunol 386:323–341. doi:10.1007/82_2014_413. 25037260 PMC4358778

[14] Krammer F. 2016. Novel universal influenza virus vaccine approaches. Curr Opin Virol 17:95–103. doi:10.1016/j.coviro.2016.02.002. 26927813 PMC4902764

[15] Angeletti D, Kosik I, Santos JJS, Yewdell WT, Boudreau CM, Mallajosyula VVA, Mankowski MC, Chambers M, Prabhakaran M, Hickman HD, McDermott AB, Alter G, Chaudhuri J, Yewdell JW. 2019. Outflanking immunodominance to target subdominant broadly neutralizing epitopes. Proc Natl Acad Sci U S A 116:13474–13479. doi:10.1073/pnas.1816300116. 31213541 PMC6612916

[16] Keating R, Hertz T, Wehenkel M, Harris TL, Edwards BA, McClaren JL, Brown SA, Surman S, Wilson ZS, Bradley P, Hurwitz J, Chi H, Doherty PC, Thomas PG, McGargill MA. 2013. The kinase mTOR modulates the antibody response to provide cross-protective immunity to lethal infection with influenza virus. Nat Immunol 14:1266–1276. doi:10.1038/ni.2741. 24141387 PMC3883080

[17] Han S, Hathcock K, Zheng B, Kepler TB, Hodes R, Kelsoe G. 1995. Cellular interaction in germinal centers. Roles of CD40 ligand and B7-2 in established germinal centers. J Immunol 155:556–567. 7541819

[18] Foy TM, Laman JD, Ledbetter JA, Aruffo A, Claassen E, Noelle RJ. 1994. gp39-CD40 interactions are essential for germinal center formation and the development of B cell memory. J Exp Med 180:157–163. doi:10.1084/jem.180.1.157. 7516405 PMC2191546

[19] Hollister K, Kusam S, Wu H, Clegg N, Mondal A, Sawant DV, Dent AL. 2013. Insights into the role of Bcl6 in follicular Th cells using a new conditional mutant mouse model. J Immunol 191:3705–3711. doi:10.4049/jimmunol.1300378. 23980208 PMC3783642

[20] Klenk HD, Garten W. 1994. Host cell proteases controlling virus pathogenicity. Trends Microbiol 2:39–43. doi:10.1016/0966-842x(94)90123-6. 8162439

[21] Venturi V, Davenport MP, Swan NG, Doherty PC, Kedzierska K. 2012. Consequences of suboptimal priming are apparent for low-avidity T-cell responses. Immunol Cell Biol 90:216–223. doi:10.1038/icb.2011.36. 21556018

[22] Bohannon C, Powers R, Satyabhama L, Cui A, Tipton C, Michaeli M, Skountzou I, Mittler RS, Kleinstein SH, Mehr R, Lee FE-Y, Sanz I, Jacob J. 2016. Long-lived antigen-induced IgM plasma cells demonstrate somatic mutations and contribute to long-term protection. Nat Commun 7:11826. doi:10.1038/ncomms11826. 27270306 PMC4899631

[23] Di Niro R, Lee S-J, Vander Heiden JA, Elsner RA, Trivedi N, Bannock JM, Gupta NT, Kleinstein SH, Vigneault F, Gilbert TJ, Meffre E, McSorley SJ, Shlomchik MJ. 2015. Salmonella infection drives promiscuous B cell activation followed by extrafollicular affinity maturation. Immunity 43:120–131. doi:10.1016/j.immuni.2015.06.013. 26187411 PMC4523395

[24] Takemori T, Kaji T, Takahashi Y, Shimoda M, Rajewsky K. 2014. Generation of memory B cells inside and outside germinal centers. Eur J Immunol 44:1258–1264. doi:10.1002/eji.201343716. 24610726

[25] Kaji T, Ishige A, Hikida M, Taka J, Hijikata A, Kubo M, Nagashima T, Takahashi Y, Kurosaki T, Okada M, Ohara O, Rajewsky K, Takemori T. 2012. Distinct cellular pathways select germline-encoded and somatically mutated antibodies into immunological memory. J Exp Med 209:2079–2097. doi:10.1084/jem.20120127. 23027924 PMC3478929

[26] Taylor JJ, Pape KA, Jenkins MK. 2012. A germinal center-independent pathway generates unswitched memory B cells early in the primary response. J Exp Med 209:597–606. doi:10.1084/jem.20111696. 22370719 PMC3302224

[27] Roco JA, Mesin L, Binder SC, Nefzger C, Gonzalez-Figueroa P, Canete PF, Ellyard J, Shen Q, Robert PA, Cappello J, Vohra H, Zhang Y, Nowosad CR, Schiepers A, Corcoran LM, Toellner K-M, Polo JM, Meyer-Hermann M, Victora GD, Vinuesa CG. 2019. Class-switch recombination occurs infrequently in germinal centers. Immunity 51:337–350.e7. doi:10.1016/j.immuni.2019.07.001. 31375460 PMC6914312

[28] Weisel FJ, Zuccarino-Catania GV, Chikina M, Shlomchik MJ. 2016. A temporal switch in the germinal center determines differential output of memory B and plasma cells. Immunity 44:116–130. doi:10.1016/j.immuni.2015.12.004. 26795247 PMC4724390

[29] Gitlin AD, von Boehmer L, Gazumyan A, Shulman Z, Oliveira TY, Nussenzweig MC. 2016. Independent roles of switching and hypermutation in the development and persistence of B lymphocyte memory. Immunity 44:769–781. doi:10.1016/j.immuni.2016.01.011. 26944202 PMC4838502

[30] Pape KA, Taylor JJ, Maul RW, Gearhart PJ, Jenkins MK. 2011. Different B cell populations mediate early and late memory during an endogenous immune response. Science 331:1203–1207. doi:10.1126/science.1201730. 21310965 PMC3993090

[31] Kurosaki T, Kometani K, Ise W. 2015. Memory B cells. Nat Rev Immunol 15:149–159. doi:10.1038/nri3802. 25677494

[32] Muramatsu M, Kinoshita K, Fagarasan S, Yamada S, Shinkai Y, Honjo T. 2000. Class switch recombination and hypermutation require activation-induced cytidine deaminase (AID), a potential RNA editing enzyme. Cell 102:553–563. doi:10.1016/S0092-8674(00)00078-7. 11007474

[33] Wrammert J, Koutsonanos D, Li G-M, Edupuganti S, Sui J, Morrissey M, McCausland M, Skountzou I, Hornig M, Lipkin WI, Mehta A, Razavi B, Del Rio C, Zheng N-Y, Lee J-H, Huang M, Ali Z, Kaur K, Andrews S, Amara RR, Wang Y, Das SR, O'Donnell CD, Yewdell JW, Subbarao K, Marasco WA, Mulligan MJ, Compans R, Ahmed R, Wilson PC. 2011. Broadly cross-reactive antibodies dominate the human B cell response against 2009 pandemic H1N1 influenza virus infection. J Exp Med 208:181–193. doi:10.1084/jem.20101352. 21220454 PMC3023136

[34] Andrews SF, Huang Y, Kaur K, Popova LI, Ho IY, Pauli NT, Henry Dunand CJ, Taylor WM, Lim S, Huang M, Qu X, Lee J-H, Salgado-Ferrer M, Krammer F, Palese P, Wrammert J, Ahmed R, Wilson PC. 2015. Immune history profoundly affects broadly protective B cell responses to influenza. Sci Transl Med 7:316ra192. doi:10.1126/scitranslmed.aad0522. PMC4770855 26631631

[35] Bajic G, van der Poel CE, Kuraoka M, Schmidt AG, Carroll MC, Kelsoe G, Harrison SC. 2019. Autoreactivity profiles of influenza hemagglutinin broadly neutralizing antibodies. Sci Rep 9:3492. doi:10.1038/s41598-019-40175-8. 30837606 PMC6401307

[36] Hoffmann E, Krauss S, Perez D, Webby R, Webster RG. 2002. Eight-plasmid system for rapid generation of influenza virus vaccines. Vaccine 20:3165–3170. doi:10.1016/s0264-410x(02)00268-2. 12163268

[37] Olsson J, Johansson J, Honkala E, Blomqvist B, Kok E, Weidung B, Lövheim H, Elgh F. 2019. Urea dilution of serum for reproducible anti-HSV1 IgG avidity index. BMC Infect Dis 19:164. doi:10.1186/s12879-019-3769-x. 30764767 PMC6376645

